# Long-term Treatment Response and Patient Outcomes for Vestibular Schwannoma Patients Treated with Hypofractionated Stereotactic Radiotherapy

**DOI:** 10.3389/fonc.2017.00200

**Published:** 2017-09-04

**Authors:** Mira A. Patel, Ariel E. Marciscano, Chen Hu, Ignacio Jusué-Torres, Rupen Garg, Arif Rashid, Howard W. Francis, Michael Lim, Kristin J. Redmond, Daniele Rigamonti, Lawrence R. Kleinberg

**Affiliations:** ^1^Johns Hopkins University, Baltimore, MD, United States; ^2^University of California, Irvine School of Medicine, Irvine, CA, United States; ^3^Drexel University College of Medicine, Philadelphia, PA, United States

**Keywords:** vestibular schwannoma, acoustic neuroma, hypofractionated radiotherapy, hearing loss, cranial nerves

## Abstract

**Purpose:**

The aim of this study is to evaluate long-term treatment outcome and toxicities among vestibular schwannoma (VS) patients treated with hypofractionated stereotactic radiotherapy (HSRT).

**Methods:**

383 patients with unilateral VS treated with HSRT (25 Gy, five fractions) between 1995 and 2007 were retrospectively reviewed. Treatment failure was defined as requiring salvage microsurgery. Posttreatment new/progressive clinical symptoms or increases in baseline tumor volume (BTV) due to treatment effect or progression were noted. Symptom outcomes were reported as baseline and posttreatment ± improvement, respectively. Symptoms were grouped by cranial nerve (CN) VII or CNVIII. Audiometry was assessed baseline and posttreatment hearing. Patients were grouped as having greater than serviceable hearing [Gardner Robertson (GR) score 1–2] or less than non-serviceable hearing (GR score 3–5) by audiometry.

**Results:**

Median follow-up was 72.0 months. Nine (2.3%) experienced treatment failure. At last follow-up, 74 (19.3%) had new/progressive symptoms and were categorized as radiologic non-responders, whereas 300 (78.3%) had no tumor progression and were grouped as radiologic responders. Average pretreatment BTV for treatment failures, radiologic non-responders, and radiologic responders was 2.11, 0.44, and 1.87 cm^3^, respectively. Pretreatment CNVII and CNVIII symptoms were present in 9.4 and 93.4% of patients, respectively. Eight (24%) with pre-HSRT CNVII and 37 (10%) with pre-HSRT CNVIII symptoms recovered CN function post-HSRT. Thirty-five (9%) and 36 (9.4%) experienced new CNVII and CNVIII deficit, respectively, after HSRT. Of these, 20 (57%) and 18 (50%) recovered CNVII and CNVIII function, respectively, after HSRT. Evaluable audiograms were available in 199 patients. At baseline and at last follow-up, 65.8 and 36.2% had serviceable hearing, respectively. Fifty-one percent had preservation of serviceable hearing at last follow-up.

**Conclusion:**

Treatment of VS with HSRT is effective with treatment success in 97.7% and an acceptable toxicity profile. Less than one-third of patients experience any new CNVII or CNVIII deficit posttreatment. Greater than 50% of patients with serviceable hearing at baseline maintained hearing function. Improved methods to differentiate treatment effect and tumor progression are needed.

## Introduction

Vestibular schwannomas (VS) are benign skull base tumors that are managed by observation, surgical resection, or radiotherapy. The optimal approach to radiotherapy is unknown and includes single-dose stereotactic radiosurgery, hypofractionated stereotactic radiotherapy (HSRT) delivered over 2–5 treatments, or conventionally fractionated stereotactic radiotherapy generally administered over 25–30 sessions (1–22). In our practice, we preferentially utilized HSRT, 25 Gy in five fractions, based on a hypothesis that this would maintain the excellent tumor control achieved with high daily dose treatment but with reduced toxicities compared to single-dose stereotactic radiosurgery.

The long-term impact of HSRT on local control, hearing, and treatment-related toxicity remains uncertain, although data from a few small cohorts suggest that the majority of patients maintain preserved serviceable hearing and low rates of facial nerve dysfunction after HSRT (1, 3–10). In addition, the association of imaging outcome with clinical endpoints has not been rigorously explored. We present long-term tumor volume progression, cranial nerve (CN) function, and audiometric data for a cohort of patients treated with HSRT.

## Materials and Methods

### Patient Selection and Stratification

After receiving institutional review board approval, the electronic medical record at a single, high volume institution was retrospectively reviewed for patients with unilateral VS receiving radiation therapy alone between 1995 and 2007. Only those who received HSRT (25 Gy in five fractions) were included. Data were collected on patient demographic information and comorbidities, tumor characteristics including laterality and baseline tumor volume (BTV), median follow-up, pretreatment CN VII or VIII deficits, posttreatment CN VII or VIII deficits, other posttreatment complications, Gardner Robertson (GR) Score by audiometric assessment at baseline and after treatment completion, and changes in tumor volume after treatment and at last follow-up (23). Patients were stratified by degree of tumor progression: patients were identified as radiologic responders if their imaging abnormality volume at last follow-up was <20% larger than their BTV, as radiologic non-responders if their imaging volume at last follow-up was ≥20% larger than their BTV, and as treatment failures if they required any kind of additional salvage therapy including microsurgery.

### Treatment Regimen

Patients received highly conformal HSRT *via* the BrainLAB Treatment Planning System (BrainLAB, Feldkirchen, Germany) and Brain Scan software, v. 5.3. Stereotactic radiation was delivered using the BrainLAB mask and frame immobilization system. The dose utilized was 500 cGy × 5 prescribed to the 80% isodose line. Contouring of treatment volume was performed using CT fused and co-registered with gadolinium contrast-enhanced T1-weighted MRI. During treatment, all patients received daily localization.

### Radiologic Evaluation

Each patient received a baseline MRI scan prior to treatment and serial MRI scans at clinically appropriate time intervals thereafter to document changes in tumor volume, measured in cubic centimeter. Tumor volume measurements were performed using the cranial–caudal, transverse, and anterior–posterior dimensions. Imaging progression or regression was documented as a percent change in volume relative to BTV.

### Landmark Analysis of Outcome

To evaluate treatment failures (need for further therapeutic intervention) as a function of time based on their tumor volume, we performed a landmark analysis. Postradiosurgery imaging outcome was assessed. Tumor volume/imaging abnormality recorded at intervals of 0–3.99, 4.0–5.99, and >6.0 years after baseline imaging. Each patient’s imaging volume was considered separately within each time interval, and they were assigned response status relative to BTV at each time point. As such, response status of one patient could change over time depending on the growth characteristics of their tumor. Patients with <20% increase in tumor volume relative to the BTV at each time interval were considered radiologic responders, those with a ≥20% increase relative to their BTV were radiologic non-responders, and those who required salvage microsurgery were treatment failures. Given the paucity of evidence regarding measures of volumetric response, we reviewed the literature, and on the basis of our clinical judgment, we decided upon a volumetric threshold of 20% above BTVs, which we felt was a conservative estimate to distinguish between progression, the error inherent in estimating volumetric changes using simple tri-dimensional measurements, and pseudoprogression (24, 25). If a patient did not have imaging within a particular time interval, they were excluded from the analysis within that time interval.

### Clinical Evaluation of Symptomatology and Hearing

The presence of CN VII or VIII deficit or other symptoms unrelated to CN VII or VIII were documented by clinical report at baseline prior to treatment and at each clinic visit posttreatment. CN VIII outcomes post-HSRT were reported separately as non-hearing and hearing outcomes with non-hearing outcomes including imbalance, tinnitus, and dizziness. Hearing outcomes were reported as a function of the Gardener-Robertson scale. Audiometric evaluation occurred prior to treatment initiation and post-HSRT. Only those patients who received audiometric evaluation prior to and after treatment were considered evaluable. Patients were grouped as having greater than serviceable hearing (GR score 1–2) or less than non-serviceable hearing (GR score 3–5) by audiometry.

### Radiation-Induced Morbidity

Treatment-related complications during follow-up including acute RT-induced neoplasm, hydrocephalus, pituitary treatment failure, and RT-related neurologic deficits were assessed and documented.

### Statistical Analysis

Categorical data were analyzed using summary statistics and presented as frequency (%). Continuous variables were summarized as mean (SD). Two-tailed *t*-test was performed to compare radiologic responders vs. radiologic non-responders with regard to BTV and percentage change in tumor volume at last follow-up (Table 2). Chi-squared test was performed to compare radiologic responders vs. radiologic non-responders with regard to posttreatment toxicities (Table 4). All statistical computations were performed using Stata version 12.1 software.

## Results

### Baseline Patient and Tumor Characteristics

A total of 446 individuals received radiation therapy for unilateral VS at our institution. Between 1997 and 2007, 383 received HSRT and had a median follow-up of 72.0 months (Table 1), and 15% (57 patients) had greater than 10-year follow-up. Prior to treatment, <5% of patients had any CN VII-related symptom, including facial twitching, facial weakness, or dysgeusia. Conversely, a significant proportion of individuals presented with CN VIII-related symptoms at baseline. Nearly all individuals had baseline subjective hearing loss (93.4%), while nearly half experienced imbalance (45.2%), and more than half suffered from tinnitus (67.9%). The most prevalent non-CN VII and VIII symptom experienced before treatment was facial paresthesia (16.4%). Fifty-two percent of tumors were right sided.

**Table 1:** Baseline patient and tumor characteristics (*n* = 383).

Age at diagnosis (mean, SD, range)	54.2 (11.4, 18–82)	
Race		
White	343 (89.6)	
Non-white	46 (12.0)	
Male	207 (54.0)	
Comorbidities	4	
Hypertension	91 (23.8)	
Diabetes	18 (4.7)	
Vascular disease	72 (18.8)	
Any smoking history	74 (19.3)	
Cranial nerve (CN) VII symptoms		
Facial twitching	16 (4.2)	
Facial weakness	14 (3.7)	
Dysguesia	6 (1.6)	
CN VIII symptoms		
Hearing loss	358 (93.4)	
Ear fullness	83 (21.7)	
Imbalance	173 (45.2)	
Tinnitus	260	(67.9)
Non-CN VII or VIII symptoms		
Facial paresthesia	63	(16.4)
Headache	37	(9.7)
Otalgia	23	(6.0)
Nausea	11	(2.9)
Vomiting	7	(1.8)
Dysphagia	0	(0)
Xeropthalmia	0	(0)
Diplopia	4	(1.0)
Alopecia	0	(0)
Fatigue	5	(1.3)
Laterality of tumor		
Left	182	(47.5)
Right	201	(52.5)
Median follow-up in months (range)	72.0	(13.6–181.5)

*Values are frequency (%) unless otherwise indicated*.

### Tumor Volume Progression

**Figure 1:**
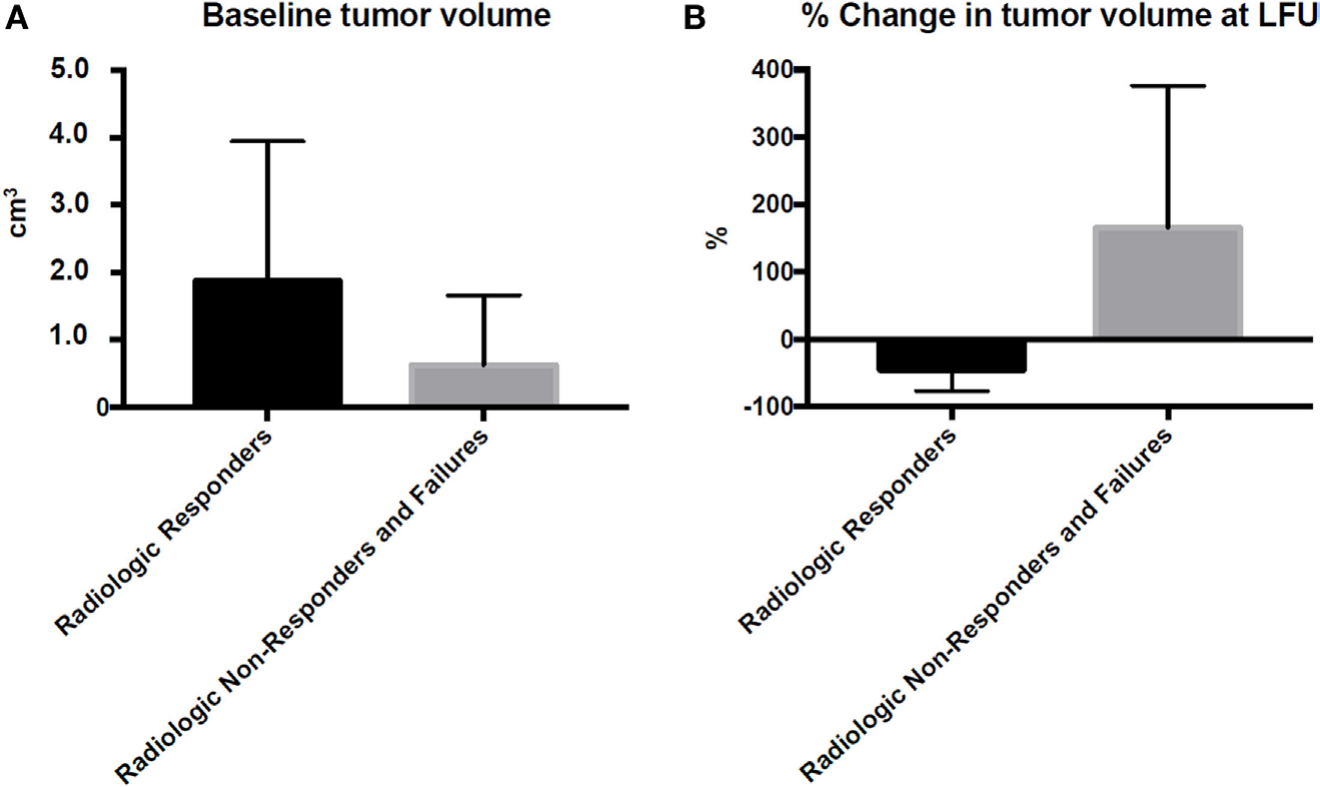
Tumor volume at baseline and change in tumor volume at last follow-up. Tumor volumetric data were gathered from MRI prior to treatment **(A)** and after treatment **(B)** at the last clinic visit. Percentage change in tumor volume at last follow-up is relative to the baseline tumor volume. LFU, last follow-up.

Based on our criteria, there were 300 radiologic responders, 74 radiologic non-responders, and 9 treatment failures within his cohort. BTV for radiologic responders, radiologic non-responders, and treatment failures was 1.87, 0.44, and 2.11 cm^3^, respectively (Table 2; Figure 1). Radiologic responders had significantly higher BTV relative to radiologic non-responders (*p* < 0.001; Table 2). While radiologic responders had an average decline in tumor volume of 45.5% of their BTV at last follow-up, radiologic non-responders and treatment failures had a 165.9 and 169.0% increase, respectively (Table 2; Figure 1). To ascertain the frequency of transient volume enlargement in the post-HSRT setting, we evaluated the volumetric changes within 2 years following HSRT. Among the 300 radiologic responders in our series, 167 patients had tumor volumetric analysis within 2 years after HSRT. Seventy-four of 167 (44%) patients demonstrated transient volumetric enlargement during this time interval. Of the radiologic non-responders, 32 had tumor volumetric analysis within 2 years after HSRT, and of those 87% had a transient increase in their tumor volume relative to baseline.

**Table 2:** Tumor volume progression.

	Radiologic responder (*n* = 300)	Radiologic non-responder (*n* = 74)	*p* Value	Treatment failure (*n* = 9)
Baseline tumor volume	1.87 (2.08)	0.44 (0.58)	<0.0001	2.11 (2.28)
Percentage change in tumor volume at last follow-up	−45.5% (31.8%)	+165.9% (210.3%)	<0.0001	+169.0% (228.1%)

*Values are average (SD) in cubic centimeter. *p* value refers to difference between radiologic responder and radiologic non-responder*.

Landmark analysis of tumor response status revealed that there was a 6% increase in the number of radiologic responders at >6 years of follow-up relative to BTV, that there were half as many radiologic non-responders at >6 years of follow-up relative to BTV, and that there were no treatment failures after 6 years (Table 3). Notably, after 6 years of follow-up, patients continued to undergo surveillance imaging every 1–2 years. Moreover, of the radiologic non-responders with imaging between 0 and 3.99 years after baseline imaging, 30 ultimately became radiologic responders at last follow-up; of the radiologic responders with imaging between 0 and 3.99 years after baseline imaging, 7 ultimately became radiologic non-responders at last follow-up, and there were 5 treatment failures who initially responded to treatment within 1.5 years after baseline imaging. There were 50 radiologic responders and 19 radiologic non-responders without follow-up within 3.99 years who were excluded in the first interval. There were 15 radiologic responders and 7 radiologic non-responders without follow-up within 5.99 years who were excluded from the first 2 time intervals.

**Table 3:** Response status at follow-up intervals measured in years from baseline imaging.

	Radiologic responder	Radiologic non-responder	Treatment failure
0–3.99 years	227 (75.7)	74 (100.0)	9 (100.0)
4.0–5.99 years	187 (62.3)	65 (87.8)	1 (11.1)
>6.0 years	241 (80.3)	44 (59.4)	0 (0)

*Time is in years after baseline imaging. Values in parenthesis are percentages of the total*.

### Pretreatment CN Symptom Resolution

Of those who presented with CNVII and CNVIII symptoms at baseline, 8 (24%) and 37 (10%), respectively, recovered affected CN function after HSRT.

### Treatment-Related CN Symptoms and Complications

Thirty-five (9%) patients experienced new CNVII deficit and 36 (9.4%) patients experienced new CNVIII deficit after HSRT (Table 4). The most common transient or permanent CNVII deficit was facial spasm (22, 6%), followed by facial weakness (5, 1.7%). At last follow-up, 3.9% had a persistent facial nerve symptom. Regarding treatment-related CNVIII non-hearing deficit, 7 (1.8%) patients had dizziness, 6 (1.6%) had tinnitus, and 5 (1.3%) had imbalance after HSRT. Of patients with treatment-related CN deficit, 20 (57%) patients recovered CNVII function and 18 (50%) patients recovered any lost CNVIII function after HSRT (Table 4).

**Table 4:** Post-HSRT symptoms and complications.

	Radiologic responder (*n* = 300)	Radiologic non-responder (*n* = 74)	*p* Value	Treatment failure (*n* = 9)	Overall (*n* = 383)
Recovery of pre-HSRT CNVII deficit	4 (16.0)	4 (50.0)		0 (0)	8 (2.1)
Recovery of pre-HSRT CNVIII deficit	32 (10.8)	5 (6.9)		0 (0)	37 (9.7)
Post-HSRT CNVII deficit	27 (9.0)	6 (8.1)	0.065	2 (22.2)	35 (9.1)
Facial weakness	5 (1.7)	1 (1.4)		0 (0)	6 (1.6)
Facial paralysis	1 (0.3)	0 (0)		0 (0)	1 (0.3)
Facial pain	1 (0.3)	0 (0)		0 (0)	1 (0.3)
Facial spasm	17 (5.7)	3 (4.1)		2 (22.2)	22 (5.7)
Dysgeusia	0 (0)	2 (2.7)		0 (0)	2 (0.5)
Post-HSRT CNVIII deficit	23 (7.7)	10 (13.5)	0.97	3 (33.3)	36 (9.4)
Hearing loss	17 (5.7)	8 (10.8)		2 (22.2)	27 (7.0)
Tinnitus	3 (1.0)	2 (2.7)		1 (11.1)	6 (1.6)
Imbalance	3 (1.0)	1 (1.4)		1 (11.1)	5 (1.3)
Dizziness	5 (1.7)	2 (2.7)		0 (0)	7 (1.8)
Recovery of post-HSRT CNVII deficit	16 (59.0)	4 (40.0)		0 (0)	20 (5.2)
Recovery of post-HSRT CNVIII deficit	10 (41.7)	7 (70.0)		1 (33.3)	18 (4.7)
Complications			0.35		
Hydrocephalus	3 (1.0)	1 (1.4)		1 (11.1)	5 (1.3)
RT-induced neoplasm	2 (0.67)	1 (1.4)		0 (0)	3 (0.8)
Pituitary failure	0 (0)	0 (0)		0 (0)	0 (0)
RT-induced neurological deficit	0 (0)	2 (2.7)		0 (0)	2 (0.5)
Any posttreatment toxicity	5 (1.7)	4 (5.4)		1 (11.1)	10 (2.6)

*Values are frequency (%). *p* value refers to difference between radiologic responders vs. radiologic non-responders with regard to symptom group*.

The most common overall posttreatment complication was hydrocephalus (five patients, 1.3%), for which one individual required ventricular shunt placement.

### Posttreatment Hearing Outcomes

There were 199 patients (52%) with evaluable hearing at baseline (radiologic responders 154, 51.3%; radiologic non-responders 40, 54.0%; treatment failures 5, 55.6%; Table 5), defined as the presence of both pretreatment and posttreatment audiometric evaluation (Table 5). One hundred thirty-one patients (66%) had greater than serviceable hearing at baseline (radiologic responders 103, 65.6%; radiologic non-responders 25, 62.5%; treatment failures 3, 60.0%; Table 5). Twenty-seven (7%) patients experienced subjective hearing loss after HSRT. By audiometric evaluation, 67 (51%) had preservation of serviceable hearing posttreatment (radiologic responders 55, 54.5%; radiologic non-responders 12, 48.0%). Sixty-two (47.3%) had a decline in functional hearing at last follow-up, defined as the progression to non-serviceable hearing at last follow-up from serviceable hearing prior to treatment initiation. At last follow-up, 72 (36.2%) had serviceable hearing. Differences in hearing outcomes between radiologic responders and radiologic non-responders were non-significant by Pearson’s Chi-squared test (*p* = 0.981).

**Table 5:** Hearing outcomes.

	Radiologic responder (*n* = 300)	Radiologic non-responder (*n* = 74)	Treatment failure (*n* = 9)	Overall (*n* = 383)
Evaluable^a^ hearing at baseline	154 (51.3)	40 (54.0)	5 (55.6)	199 (52.0)
Serviceable hearing at baseline	103 (66.9)	25 (62.5)	3 (60.0)	131 (65.8)
Preservation of serviceable hearing post-HSRT	55 (53.4)	12 (48.0)	0 (0)	67 (51.1)
Functional decline^b^ in serviceable hearing post-HSRT	46 (44.7)	13 (52.0)	3 (100.0)	62 (47.3)
Serviceable hearing at last follow-up	58 (37.7)	14 (35.0)	0 (0)	72 (36.2)

*Values are frequency (%) unless otherwise indicated*.

*^a^Evaluable hearing refers to those with pretreatment and posttreatment audiometric evaluation*.

*^b^Functional decline refers to the progression to non-serviceable hearing at last follow-up from serviceable hearing at baseline. χ^2^, p > 0.05 between radiologic responders, non-responders, and treatment failures*.

*GR, Gardner Roberston*.

## Discussion

Our findings demonstrate that HSRT is an appropriate treatment modality for unilateral VS, with an uncommon need for further intervention, low posttreatment CNVII and CNVIII toxicity, and hearing preservation in greater than half of treated patients.

Recent studies regarding the efficacy of HSRT for the treatment of VS demonstrate local control rates of 92–100% (5–7, 9, 10), with control defined as achieving the objective of no further treatment requirements. Similarly, in our cohort, we had a local control rate of 98%—as defined by the lack of need for surgical intervention—with radiologic responders having an average decrease in tumor volume of 45.5% at last follow-up. Relative to stereotactic radiosurgery (SRS) for VS, HSRT results in comparable intervention-free local tumor control (26–29). In a large series of 839 patients treated with gamma knife radiosurgery that established the role of single-dose stereotactic therapy as an important treatment for this illness, control rate was 97% defined as no need for further intervention (30). Furthermore, similar to studies of SRS treatment of VS, in this cohort, there were no new post-HSRT treatment failures after 4–6 years (15).

The long-term durability of control after radiosurgery remains an important question, especially as the alternative of surgery provides durable control albeit with a different risk profile. We did not identify any treatment failures later than the first 6 years of follow-up, suggesting that HSRT may result in durable control. This is emphasized by the fact that patients continued to have imaging follow-up every 1–2 years even 6 years after baseline imaging. This is similar to the long-term results reported by other investigators that suggest that late treatment failure is uncommon after single-dose radiosurgery (15, 29). Hasegawa et al. reported results of 440 patients with median follow-up of 12.5 years and observed 12 treatment failures within 3 years of therapy, 8 treatment failures >3 years after therapy, and no treatment failures after 10 years (29). In another large series from the University of Pittsburgh, no treatment failures were observed after 4 years (15). Interestingly, we observed that transient volumetric enlargement of tumors in the initial years after treatment did not necessarily predict treatment failure, as a number of these individuals ultimately did not demonstrate further imaging progression but actually became radiologic responders.

Indeed, imaging outcome may not be a clear measure of treatment success given radiation-related imaging changes and the difficulties of accurate determination of volume by bidimensional measurement of relatively small tumors. For this reason, we propose that long-term avoidance of need for further tumor therapy, although subject to some bias, and not imaging stability is the ultimate goal and should be the metric for treatment success. The imaging outcome reaffirms that enlargement of abnormality after radiosurgery for VS may only represent a progressive process that will require intervention in a small proportion of patients. Reports of outcome emphasize either lack of need for further intervention or imaging control as the outcome measure, without standard definitions (31). Of note, 44% of responders in our cohort had transient tumor volumetric enlargement after HSRT, which may have resulted in temporary CNVII and CNVIII symptoms that ultimately resolved with tumor regression. In addition, although 21.7% of patients had enlarged imaging abnormality at last follow-up, only 1.8% of patients required intervention for a progressive process suggestive of tumor growth. This has been demonstrated in other studies reporting imaging outcome where the majority of patients with imaging enlargement did not require further intervention for recurrent tumor, and treatment failure was defined as progressive growth without stabilization of tumor volume (15, 27). Boari et al. in reviewing the literature emphasized that for 15–30% of cases, there is a transient increase in tumor volume; for 5–10% of cases, there is stabilization after increase in tumor volume; and for <5% of cases, continuous growth is considered to be true progression (27). Although very long-term follow-up is required to confirm the significance of enlargement in this patients, we hypothesize that there may be both temporary and permanent radiosurgery-related imaging changes.

The majority of patients in recent studies experience no permanent damage to CN function after HSRT (5, 9, 32, 33). In one study, all patients with VS treated with HSRT had total preservation of long-term CN function; in another, no permanent trigeminal nerve or facial nerve toxicity was observed in any patient as a result of treatment, and in a third study of 60 patients with VS treated with HSRT, there were no severe treatment-related CN complications, and less than 15% of patients experienced any new CN symptom after treatment (5, 9, 34). Only 1.7% demonstrated a facial nerve palsy or paralysis, similar to other reports (Table 6). Indeed, Meijer et al. demonstrate that the 5-year preservation probability of facial nerve function after HSRT is near 100% (6). Similarly, we found that in the 9% who had new CNVII and 9.4% who had new CNVIII deficits after HSRT, 57 and 50% of those individuals, respectively, recovered CN function (Table 4). In this retrospective study, we could not assess the severity of the deficits.

**Table 6:** CN toxicity with HSRT vs. SRS: review of the literature.

Reference	*n*	CN VII toxicity	Hearing preservation
**HSRT**			
Hansasuta et al. (32)	383	0.2%	76%
Tsai et al. (35)	117	N/A	81.5%
Vivas et al. (36)	73	N/A	53.5%
Vernimmen et al. (33)	51	8.3%	42%
Anderson et al. (3)	37	2.1%	63.2%
**SRS**			
Breivik et al. (28)	113	12%	36%
Hasegawa et al. (29)	440	2.2%	43% 5 years, 34% 8 years
Boari et al. (27)	379	1.1%	49%
Anderson et al. (3)	48	2.1%	60%

*HSRT, hypofractionated stereotactic radiotherapy; SRS, stereotactic radiosurgery; n, number of patients; CN, cranial nerve*.

Hearing preservation is a topic of great concern after radiation therapy for VS, particularly given the close proximity of the structures of hearing to the planning target volume of radiation. The use of hypofractionated stereotactic treatment as an alternative to single-dose stereotactic radiosurgery was driven by the goal of taking advantage of the greater therapeutic ratio observed with fractionation with regard to hearing preservation. Fractionated SRT provides the benefit of delivering a lower dose of radiation during each treatment visit to such critical structures, rather than the high-dose radiation delivered by SRS. Thus far, the majority of studies of hearing preservation after HSRT demonstrate that greater than half of patients have preserved serviceable hearing (42–81%; Table 6) (3, 9, 10, 32, 33, 35–37), although gradient deterioration of pure tone averages has been observed even in those with hearing preservation (7). Hayden Gephart et al. comment that a higher radiation dose and larger cochlear volume within the radiation field result in poorer hearing outcomes posttreatment (37). Following this logic, the delivery of a high total dose of radiation divided into few low-dose fractions *via* HSRT would improve hearing outcomes relative to SRS. Indeed, HSRT has demonstrated improved hearing preservation relative to SRS in a number of studies (42–81.5% preserved hearing vs. 36–60%; Table 6) (27–29, 32, 33, 35, 36). Our results corroborate previous findings, as we observed preservation of serviceable hearing in 51% of patients after HSRT (Tables 5 and 6). This result is similar to the hearing preservation observed with stereotactic radiosurgery and does not support superiority of hypofractionated stereotactic treatment (Table 6). Overall, less than 5% of patients in our cohort continued to experience treatment-related CNVII and CNVIII deficit beyond our follow-up time of 6 years, which is consistent with the current literature regarding functional CN outcomes after HSRT and is comparable to CN outcomes after SRS (15).

A weakness of this study is that it is a retrospective analysis of patients from a wide geographic base such that a significant proportion were lost to follow-up as they ceased care and follow-up at the treating facility. Indeed, large series reporting results of therapeutic options for VS are retrospective, and comparing results should be done with caution and understanding of the limitations. Nevertheless, this report of outcome for a large cohort of patients treated with HSRT does not provide evidence to support the hypothesis that control or toxicity is superior to the outcome achieved with standard single-dose radiosurgery. This study represents results with this treatment approach of hypofractionated stereotactic radiation, and as such there is no direct comparison group; the results must be interpreted in the context of results at other institutions with this limitation in mind.

We show that HSRT is an appropriate treatment modality for unilateral VS, with a high tumor control rate, low CN toxicity, and acceptable hearing preservation relative to prior studies of SRS. However, the results do not appear to confirm that hearing preservation and toxicity could be superior to the outcome with standard single-dose radiosurgery, and we have reverted to that approach for lesions meeting appropriate size criteria. Continued research to document long-term outcomes and to reduce treatment morbidity is required. Research directions may be directed at better selection of those who require treatment rather than observation as well as use of other radiotherapy schemas that potentially may result in reduced toxicity, including hearing loss.

## Author Contributions

MP and AM: data gathering, analysis, and manuscript preparation. IJ-T, AR, and RG: data gathering and analysis. CH: data analysis and statistical methodology. HF, ML, and DR: data gathering. KR: data gathering, analysis, and manuscript review. LK: research conception, data gathering and analysis, and manuscript preparation.

## Conflict of Interest Statement

The authors declare that the research was conducted in the absence of any commercial or financial relationships that could be construed as a potential conflict of interest.
